# Temporal order of diagnosis between gambling disorder and substance use disorders: Longitudinal results from the Norwegian Patient Registry

**DOI:** 10.1016/j.abrep.2023.100501

**Published:** 2023-06-04

**Authors:** Lisa-Christine Girard, Mark D. Griffiths, Ingeborg Rossow, Tony Leino, Anna E. Goudriaan, Otto R.F. Smith, Ståle Pallesen

**Affiliations:** a Department of Psychosocial Science, University of Bergen, Norway; b Department of Psychology, Nottingham Trent University, UK; c Department of Alcohol, Tobacco and Drugs, Norwegian Institute of Public Health, Norway; d Department of Health Promotion, Norwegian Institute of Public Health, Norway; e Department of Psychiatry, Amsterdam UMC Location University of Amsterdam, Netherlands; f Amsterdam Public Health, Mental Health, Amsterdam, Netherlands; g Department of Teacher Education, NLA University College, Norway; h Norwegian Competence Centre for Gambling and Gaming Research, University of Bergen, Norway; i Department of Special Needs Education, University of Oslo, Norway

**Keywords:** Gambling disorder, Substance use disorders, Co-occurrence, Registry data, Longitudinal

## Abstract

**Introduction:**

Previous research has established co-occurrence between substance use disorders (SUDs) and gambling disorder (GD). Less well understood is the temporal sequencing of onset between these disorders, and in particular whether SUD is a risk factor for GD. The present study examined the temporal order between registered diagnoses of SUD and GD, stratified by sex.

**Methods:**

A study with a longitudinal design using objective registry data drawn from the Norwegian Patient Registry was carried out. Among the patients with a registered diagnosis of GD between 2008 and 2018 (*N* = 5,131; males = 81.8%), those (who in addition) had a registered diagnosis of any SUD (*n* = 1,196; males = 82.1%) were included. The measures included a registered diagnosis using the ICD-10 of both GD (code F63.0) and SUDs (codes F10-F19) by a health care professional. Binomial tests were used to identify the temporal order between SUD(s) and GD. Co-occurring cases (i.e., cases diagnosed within the same month) were removed in the main analyses.

**Results:**

Results showed a significant directional path from SUD to GD but no support for the reversed path (i.e., from GD to SUD). This finding was similar overall for (i) both males and females, (ii) when different SUDs (alcohol, cannabis, sedatives, and polysubstance) were examined individually, and (iii) when specifying a 12-month time-lag between diagnoses.

**Conclusions:**

The findings suggest that experiencing SUD(s) is a risk marker for GD given the temporal precedence observed for patients in specialised healthcare services seeking treatment. These results should be considered alongside screening and prevention efforts for GD.

## Highlights

• Substance use disorder (SUD) is a risk marker for gambling disorder (GD)
• Temporal order between SUD and GD does not differ for males and females.
• A previous history of SUD should be accounted for in screening and prevention of GD.

## 1 Introduction

Gambling disorder (GD) is a clinical diagnosis characterized by frequent and continual engagement in gambling activities, resulting in detrimental impacts across family, social, economic, and occupational domains of living (ICD-10, 2016). Problem gambling is similarly characterized but in the absence of meeting criteria for a formal diagnosis (Potenza et al., 2019). Both the disorder and problem gambling are considered serious public health issues given their impact not just for the individual involved but also for their personal network (e.g., an estimated 5 to 15 people are affected for every one individual with GD/gambling problems; Kalischuk et al., 2006). Global reported prevalence rates vary by country (i.e., 0.1%-5.8%), although direct comparisons present challenges given methodological differences (for a comprehensive review, see Calado and Griffiths (2016)). In Norway (where the present study was carried out), the prevalence of self-reported gambling problems in 2019 was 1.4%, which increased from 0.9% in 2015 (Pallesen et al., 2020).

In the context of increasing prevalence, there is a high importance placed on the identification of temporal risk markers associated with GD. One such risk marker may be substance use disorder (SUD). Notably, a substantial body of literature has identified that there is a significant co-occurrence of SUDs (e.g., alcohol, drugs, tobacco) among individuals with GD (e.g., Lorains et al., 2011, Yakovenko and Hodgins, 2018). Estimates of co-occurrence are typically highest for nicotine dependence, reaching upwards of 60%, and lowest for drug use disorders (DUDs), leaving alcohol use disorders (AUD) in between (often around 20%-30 %) (Lorains et al., 2011, Rash et al., 2016). The literature has identified several shared risk and etiological factors of both GD and SUDs including being male, young age, deprived socio-economic background, somatic and mental illness, as well as genetics (Dowling et al., 2017, Merikangas and McClair, 2012, Potenza et al., 2019, Yau and Potenza, 2015).

While co-occurrence is recognized, there are few studies that have delineated the temporal sequencing between GD or problem gambling and SUDs using individual-level data (Yakavenko & Hodgins, 2018), and mixed findings have been reported regarding the type of SUD involved. For example, using a large cross-sectional nationally representative face-to-face household survey from the US (*n* = 3,435), Kessler et al., (2008) reported that GD largely preceded tobacco dependence, whereas alcohol or drug abuse preceded GD. On the other hand, in another cross-sectional US-based study also using face-to-face interviews, Cunningham-Williams et al. (2000) reported that cannabis dependence preceded GD in a combined sample of drug treatment-seekers and a high-risk drug user non-treatment sample (*n* = 990), although in the specific case of stimulants (i.e., cocaine or amphetamines), this type of SUD was more likely to follow GD. Moreover, in a cross-sectional study using an Australian sample of treatment-seeking problem gamblers (*n* = 267), Haw and Holdsworth (2016) reported that for all three categories of self-reported SUDs (alcohol, nicotine, drugs), these were most often precursors to problem gambling. Taken together, these findings – which are all based on self-reported onset of *both* problem gambling and SUD – seem to suggest some mixed patterns of temporality that may be dependent on particular factors, including the type of population sampled (e.g., general population, treatment-seekers) and type of SUD. Noteworthy, DUD (i.e., cannabis use) and AUD have consistently been found to precede GD across these studies.

This limited literature offers little regarding possible differences in temporality with regard to sex differences. However, findings using cross-tabulated census data from the Norwegian Patient Registry, suggest sex differences in timing of SUD for patients with GD (Leino et al., 2021). More specifically, males with GD had a higher risk of a SUD diagnosis within the same year whereas females with GD had a higher risk of diagnosis of SUD one or more years after initial onset of GD. This study also found SUD to be more prevalent among individuals with GD (i.e., 22.5%), as compared to GD among individuals with SUD (i.e., 0.7%). While the Leino et al. (2021) study use the same registry data as the current study to investigate SUD and GD, the studies differ in terms of their aim, scope, and research questions. For example, Leino et al. (2021) used aggregate level data and did not undergo any inference-based statistical analysis, whereas the present study examines individual level data using inferential analysis. The time span is also longer in the present study, and we further distinguished between type of SUD experienced.

It should also be noted that methodological factors pose major challenges for the identification of temporality and the interpretation of previous findings. As aforementioned, common in the literature is the use of retrospective cross-sectional studies asking participants to recall the onset or occurrence of symptomology concerning both GD and SUDs. Moreover, the use of subjective data (i.e., self-report data from surveys and interviews) rather than the use of objective data (e.g., medical records and registry data), is most often employed. Self-report data, in combination with cross-sectional retrospective designs, enhances the potential risk for introducing multiple forms of bias, including recall bias, social desirability, and differential recall (Coughlin, 1990). Shared method variance bias is also of concern. This makes it challenging to accurately delineate the temporal order between onset of disorders. Consequently, prospective studies using objective data are needed to enhance the rigor in this emerging area of work and to further add to the understanding of which type of disorder is more likely to precede the other.

### 1.1 Aims of the present study

Using national health registry data, the present study aimed to better delineate the temporal order of the association between GD and SUDs, in particular examining whether SUD is a risk marker for GD. In addition, the aim was to examine whether there were any differences in the direction of temporal order between (i) females as compared to males, (ii) between individual SUDs (i.e., alcohol, cannabis, sedatives, polysubstance), and (iii) when accounting for a 12-month time lag between diagnoses. These additional aims were important given the literature suggesting sex differences in timing of risk of SUD among patients with GD (Leino et al., 2021), and that the direction between GD and SUD may differ as a function of the type of SUD in question (e.g., Afifi et al., 2016, Cunningham-Williams et al., 2000).

## 2 Methods

### 2.1 Participants and design

Using a longitudinal design, patients were drawn from the Norwegian Patient Registry (NPR) (Bakken et al., 2020), a nationwide registry owned by the Norwegian Directorate of Health, which includes all admissions to specialized health care in publicly funded hospitals/clinics. Detailed health information is collected in the registry concerning all disease diagnoses using the *International Classification of Diseases*, *10th Revision* (ICD-10), dates of all disease diagnoses made (month and year), hospital stays, inpatient and outpatient treatment received, the patient’s social security number, sex, and age, among others, for all residents of Norway. Health information collected in the registry between 2008 and 2018 was used as a result of the registry having complete data on patients from 2008. The end date was set to 2018 because the application for data access was made in 2019. Inclusion criteria in the present study was having a registered diagnosis of GD (code F63.0 in the ICD-10) between 2008 and 2018 (*n* = 5,131). Additionally, patients with GD also had to have at least one registered diagnosis of SUD (i.e., codes F10-F19: alcohol-related disorders; opioid-related disorders; cannabis-related disorders; sedative- or hypnotic-related disorders; cocaine-related disorders; other stimulant-related disorders; hallucinogen-related disorders; nicotine dependence; inhalant-related disorders; and polysubstance or other psychoactive substance-related disorders) recognized in the ICD-10, between 2008 and 2018 (*n* = 1,169). The prevalence rates of co-occurrence of individual SUDs for the entire sample of patients with GD are presented in Table 1. The final sample comprised 1,169 patients with both a diagnosis of GD and SUD, 960 of whom were males (82.1%). The mean age of patients was 41.7 years, with a standard deviation of 11.5 years. To classify the order of diagnosis, the first registered date (i.e., month and year) of diagnosis in the registry was used. However, there was no information regarding the specific day of the month for any diagnosis in the registry. Ethics approval was granted by the Regional Committee for Medical and Health-Related Research Ethics in Western Norway (no. 30393). All data were anonymized by the Directorate of Health prior to receiving and handling the data.

**Table 1:** Prevalence of co-occurrence of ICD-10 diagnosis for patients with GD.

Diagnostic categories	% (*n*)
SUD (combined F10-F19)	22.8% (1,169)
Alcohol	14.6% (748)
Opioids	2.4% (123)
Cannabis	5.7% (291)
Sedatives	4.8% (245)
Cocaine	1.7% (89)
Stimulants	3.7% (190)
Hallucinogens	0.4% (18)
Nicotine	0.7% (37)
Inhalants	0.2% (8)
Polysubstance use	6.5% (331)

*Note. N* = 5,131, all patients with a registered diagnosis of GD.

### 2.2 Statistical analysis

The predominant temporal order (i.e., which disorder predominantly occurs before the other in the register) was examined using binomial probability tests for each direction (one-tailed). The analyses first examined the temporal order between any SUD diagnosis (i.e., codes F10-F19 combined) and GD. Next, the analyses were conducted looking at pairs of individual F10-F19 codes with GD, that were sufficiently powered (i.e., F10, F12, F13 and F19). Power was calculated for a binomial test assuming *p* = 0.4 for proportion 1 and *p* = 0.6 for proportion 2 with power and alpha set at 0.95 and 0.05 respectively, requiring a sample size of 67. The study also conducted all analyses accounting for a 12-month lag between diagnoses (i.e., only considering directionality when diagnosis occurred more than 12 months apart). The probability of success was set to equal 0.5 (implying that there is at least a 50% probability that one specified disorder precedes the other disorder) given the limited number of previous studies formally examining temporal order and the mixed findings reported. Success was defined as the occurrence of a diagnosis of SUD (combined F10-F19), or individual F10, F12, F13 and F19 codes as the first disorder given the main interest in examining whether SUD is a risk marker for GD. Patients with a diagnosis of co-occurring onset (same month and year) in the registry were excluded from the main analysis of temporal order because there was no reliable means of determining which disorder came first, and since a time lag of less than one month is not reliable in terms of studying substantial temporality. However, supplementary analyses were also conducted using two binomial tests (i.e., SUD as the first diagnosis as compared to the binomial distribution of the sample size: N_SUD_ + N_co-occurring_ + N_GD_ and then GD as the first diagnosis as compared to the binomial distribution of the sample size: N_SUD_ + N_co-occurring_ + N_GD_), which are presented in the Supplementary Material (eTables 1–2). The analyses further examined whether temporal order in pairs of disorders differed as a function of sex, although in the case of cannabis-related disorders it was not possible to examine the temporal order in women due to limited power (i.e., only 10 patients). Confidence intervals were calculated using the exact Clopper-Pearson calculation. All analyses were conducted in Stata v17.0. Hereafter, the term ‘significance’ is used *in lieu* of statistical significance.

## 3 Results

### 3.1 Temporal order

Results of the binomial probability test examining any SUD diagnosis (i.e., codes F10-F19 combined) with GD showed an observed probability of 0.65 (95% CI: 0.62–0.68) for SUD as the first diagnosis. The directional test from SUD to GD was significant (i.e., *n* = 907, *p* <.001). Results examining AUD (F10) and GD showed an observed probability of 0.62 (95% CI: 0.57–0.66) for AUD as the first diagnosis. The directional test from AUD to GD was significant (i.e., *n* = 427, *p* <.001). Results examining cannabis-related disorders (F12) and GD showed an observed probability of 0.74 (95% CI: 0.66–0.80) for cannabis-related disorders as the first diagnosis. The directional test from cannabis to GD was significant (i.e., *n* = 167, *p* <.001). Results examining sedative-related disorders (F13) and GD showed an observed probability of 0.69 (95% CI: 0.58–0.78) for sedative-related disorders as the first diagnosis. The directional test from sedatives to GD was significant (i.e., *n* = 89, *p* <.001). Finally, results examining polysubstance-related disorders (F19) and GD showed an observed probability of 0.71 (95% CI: 0.63–0.78) for polysubstance-related disorders as the first diagnosis. The directional test from polysubstance to GD was significant (i.e., *n* = 89, *p* <.001) (see eFigure 1 in Supplementary Material). The same patterns of temporal order were observed when stratified by sex (see Table 2, and eFigures 2–3), and when accounting for a 12-month lag between diagnoses (see Table 3, and eFigures 4–6). Sensitivity analyses including the entire sample (i.e., co-occurring cases included) are presented in the Supplementary Material (eTables 1–2, eFigures 7–12), which generally support the main findings of the predominant direction. However, these sensitivity analyses suggest no preferred direction between GD and SUD when accounting for a 12-month period between the first and second diagnosis.

**Table 2:** Temporal order of pairwise disorders for males and females: SUD(s) and GD.

SUD	*n*	Observed probability	95% CI	Directional test of significance from SUD to GD	Directional test of significance from GD to SUD
SUD (F10-F19 combined)					
Males	734	0.64	0.60–0.67	*p* <.001	*p* = 1.000
Females	173	0.71	0.63–0.77	*p* <.001	*p* = 1.000
Alcohol (F10)					
Males	391	0.61	0.56–0.66	*p* <.001	*p* =.999
Females	81	0.64	0.53–0.75	*p* =.007	*p* =.996
Cannabis (F12)					
Males	157	0.72	0.64–0.79	*p* <.001	*p* = 1.000
Females	10	–	–	*–*	*–*
Sedatives (F13)					
Males	54	0.69	0.54–0.80	*p* =.004	*p* =.998
Females	35	0.69	0.51–0.83	*p* =.020	*p* =.991
Polysubstance (F19)					
Males	115	0.70	0.61–0.79	*p* <.001	*p* =.999
Females	32	0.72	0.53–0.86	*p* =.010	*p* =.996

*Note*: Co-occurring cases were first removed from each pairwise analysis, this included 22.4% of cases from SUD (combined F10-F19), 24.2% of cases from F10, 21.2% of cases from F12, 18.4% of cases from F13, and 19.7% of cases from F19.

**Table 3:** Temporal order of pairwise disorders: SUD(s) and GD with a 12-month lag.

SUD	*n*	Observed probability	95% CI	Directional test of significance from SUD to GD	Directional test of significance from GD to SUD
SUD (F10-F19 combined)	727	0.66	0.62–0.69	*p* <.001	*p* = 1.000
Males	587	0.65	0.61–0.69	*p* <.001	*p* = 1.000
Females	140	0.70	0.62–0.77	*p* <.001	*p* = 1.000
Alcohol (F10)	380	0.63	0.58–0.68	*p* <.001	*p* = 1.000
Males	316	0.62	0.56–0.67	*p* <.001	*p* =.999
Females	64	0.66	0.53–0.77	*p* =.008	*p* =.995
Cannabis (F12)	142	0.75	0.67–0.82	*p* <.001	*p* = 1.000
Males	132	0.73	0.64–0.80	*p* <.001	*p* = 1.000
Females	10	–	–	*–*	*–*
Sedatives (F13)	73	0.70	0.58–0.80	*p* <.001	*p* =.999
Males	46	0.70	0.54–0.82	*p* =.005	*p* =.997
Females	27	0.70	0.49–0.86	*p* =.026	*p* =.990
Polysubstance (F19)	110	0.73	0.63–0.81	*p* <.001	*p* = 1.000
Males	86	0.74	0.64–0.83	*p* <.001	*p* =.999
Females	24	0.67	0.45–0.84	*p* =.043	*p* =.077

*Note*: Cases where the first diagnosis occurred within 12 month of the second diagnosis were considered as co-occurring. Co-occurring cases were removed from each pairwise analysis, this included 37.8% of cases from SUD (combined F10-F19), 39.0% of cases from F10, 33.0% of cases from F12, 33.0% of cases from F13, and 39.9% of cases from F19.

## 4 Discussion

The overarching aim of the present study was to better understand the temporal order between SUD and GD among GD patients, in particular whether SUD is a risk marker for GD, across different specifications (e.g., sex, type of SUD, and accounting for a 12-month period between diagnoses). Although there are numerous studies which have identified co-occurrence between SUD and GD as commonplace, the present study is of importance as there are few studies which have delineated the temporal sequencing of co-occurrence between them.

Using objective population registry data, the results of the temporal sequencing analyses suggested that a diagnosis of SUD typically predates, and is therefore a risk marker, for a diagnosis of GD within the Norwegian treatment-seeking population, irrespective of type of SUD examined. No support was found for the inverse direction of GD being a potential risk factor for a subsequent diagnosis of SUD. This same temporal pattern was also observed when the analyses were stratified by sex, suggesting that at least with respect to sequencing, patterns were similar among males and females. To date, the relatively few studies that have examined the temporal order between GD and SUD have produced somewhat mixed findings.

Support has been found for the directional path from GD to SUD only with regard to nicotine dependence (Kessler et al., 2008), whereas the inverse path from various types of SUD to GD or gambling problems is more often reported (Afifi et al., 2016, Cunningham-Williams et al., 2000, Haw and Holdsworth, 2016). The results of the present study are largely in line with these latter studies suggesting directional paths of diagnosis are more often from SUD to GD than the other way around. However, unlike in these previous studies, the directional paths were not conditional in the present study and were independently consistent (i) for the entire sample, (ii) when broken down and examined individually by type of SUD (i.e., F10, F12, F13 and F19), (iii) for males and females, and (iv) when accounting for a 12-month lag between diagnoses.

Differences in samples and methodology could in part account for the differences in findings across all studies. For example, the use of surveys from the general population as compared to participants who had initiated help-seeking behaviours (i.e., having a registered diagnosis from a medical professional or in treatment), along with high reliance on retrospective self-reports of both disorder and onset, as compared to the use of prospective health registry data, such as in the present study. Therefore, the findings add to the small but growing literature examining temporal order between GD and SUD, suggesting that within the Norwegian treatment-seeking population, SUD is a risk marker for GD rather than GD being a risk marker for SUD. A possible explanation for the temporal order revealed in the present study is that maladaptive gambling for some may act as a way of escaping (Neophytou et al., 2021), for instance, from the negative consequences of various chemical addictions. Alternatively, it is also possible that gambling may act as a substitution for some SUD patients who have managed to quit their drug habit. Neither possibility can be confirmed or dismissed given the data in the present study. However, a better understanding of why the temporal order observed occurs is an important next step for researchers in the field. A mixed-methods approach would help in better understanding this evolution of symptoms from SUD to GD.

These results might then suggest that among SUD patients, screening for GD and referral to counselling or treatment may be appropriate given the co-occurrence between the two. Currently, approximately 40% of the clinics in Norway offering specialist treatment care for SUD also offer specific treatment for gambling problems, with just over 20% of them further screening for gambling problems among all new patients, irrespective of the reason for referral. Clinics offering treatment for gambling problems were found to be present in all health regions in Norway (Torvund et al., 2018). Increasing these screening rates even further appear to be warranted in the context of the present study’s findings. Additionally, treatment efforts for SUD patients may be well placed to focus on strategies that aim to inhibit nonsubstance-related addictive behaviours such as GD, which are not homogenous to SUD, but which may in part share several psychopathological risk factors with SUD (e.g., genetic vulnerability, early life stress). Underlying decision-making processes, motivation, and pleasure/reward processing may be targets for both treatment of SUD and prevention of GD among this population (Yau & Potenza, 2015). On the other hand, it is possible that the results are in part driven from patients seeking earlier treatment of SUD as compared to GD, due to more severe or intolerable sequelae experienced. Earlier treatment of SUD may then have led to the subsequent identification of GD and its consequent treatment. As the analysis is based on the main diagnosis in specialised treatment, this interpretation cannot be ruled out and warrants further attention in future studies.

### 4.1 Strengths and limitations

There are several noteworthy strengths of the present study, including the use of prospective and objective population-based registry data spanning over a whole decade, free from recall, non-response, and other self-reporting biases. Moreover, as Norwegian specialized treatment services are virtually free of charge, there is little systematic selection to treatment due to economic barriers. Furthermore, the sample size was reasonably high. Despite these strengths, some inherent limitations must be considered. First, only patients under specialised care are included in the registry (i.e., those with a diagnosis from a health professional). Arguably, these individuals may differ from those who do not seek help and are not included within the registry, which are known to represent the vast majority of those with gambling problems (Ladouceur, 2005). Relatedly, there may be differences in health seeking behaviour across different types of SUDs, differences in screening practices across SUDs, which may increase or decrease the likelihood of diagnoses, and differences in treatment coverage in specialized health services across specific SUD diagnoses (Amundsen et al., 2022), which may result in findings being less representative of the co-occurrence of various SUDs among GDs (e.g., nicotine dependence). Differences in prevalence rates have also been observed between younger and older age groups based on survey literature as compared to clinical studies in primary health care and from registry studies of admissions to specialized health care (i.e., there is a higher prevalence of older patients among the latter). Thus, generalisability of the study findings outside of treatment-seeking individuals warrants caution. Second, because there was no information related to the exact day of diagnosis, only the month and year, a substantial number of cases were excluded from the main analysis of temporal order (i.e., all co-occurring cases). However, while sensitivity analyses (presented in the Supplementary Material) generally supported the main findings in the results, the directional probability may be somewhat under-estimated or over-estimated. Third, the data are limited to diagnoses that occurred between 2008 and 2018. Therefore, it cannot be completely ruled out that any previously remitting diagnosis could have resulted in bias in the identification and classification between the first and second disorder. Moreover, in the present study, no information was available regarding the duration of problem behaviour prior to receiving a formal diagnosis for GD or SUD. Fourth, given that participants in the present study were selected from the registry on the basis of a diagnosis of GD, detection and/or surveillance, bias may be present. This may have resulted in an overestimation of the association between GD and SUD. Fifth, socio-demographic (e.g., employment, family status), health, and psychological factors (such as mood disorders) that may be important in understanding the association between SUD and GD, were not accounted for in the present study. Future studies could build on the findings presented here by considering the role of these factors. Finally, the low prevalence of females in the sample resulted in an inability to examine the temporal order between GD and specific SUDs (e.g., cannabis). This presents a knowledge gap that future studies should address.

## 5 Conclusions

In the context of these limitations, the present study’s results provide a rigorous overview of temporal order strongly suggesting that among patients in specialized health services, SUD precedes and is a risk marker for GD, both in the case of males and females, and for all individual types of SUDs examined (i.e., alcohol, cannabis, sedatives, and polysubstance use). Taken together, these results suggest possible actionable targets for intervention and prevention programs, for instance, improving screening/early detection for GD in SUD treatment, and addressing cross-addiction – including behavioural addictions like GD – during SUD treatment. As the study population consisted of those with GD in specialised care, there is still a need for population-based research to examine the association between gambling problems and SUDs because many of those with gambling problems or SUD do not seek treatment.

## Financial Disclosure Statement

The present study was funded by the Research Council of Norway, grant no. 273718. The authors have no other financial relationships relevant to this article to disclose.

## CRediT authorship contribution statement

**Lisa-Christine Girard:** Conceptualization, Methodology, Writing – original draft, Writing – review & editing, Visualization. **Mark D. Griffiths:** Writing – review & editing. **Ingeborg Rossow:** Conceptualization, Writing – review & editing. **Tony Leino:** Data curation, Writing – review & editing. **Anna E. Goudriaan:** Writing – review & editing. **Otto R.F. Smith:** Methodology, Writing – review & editing. **Ståle Pallesen:** Conceptualization, Methodology, Writing – review & editing, Supervision, Project administration, Funding acquisition.

## Declaration of Competing Interest

The authors declare the following financial interests/personal relationships which may be considered as potential competing interests: ‘The authors (except MDG) have no conflicts of interest relevant to this article to disclose. MDG has received research funding from *Norsk Tipping* (the gambling operator owned by the Norwegian government). MDG has received funding for a number of research projects in the area of gambling education for young people, social responsibility in gambling and gambling treatment from Gamble Aware (formerly the Responsibility in Gambling Trust), a charitable body which funds its research program based on donations from the gambling industry. MDG undertakes consultancy for various gambling companies in the area of social responsibility in gambling.’

## Data Availability

The authors do not have permission to share data.
