# Supplementary material for: Temporal order of diagnosis between gambling disorder and substance use disorders: Longitudinal results from the Norwegian Patient Registry
Source: Addict Behav Rep. 2023 Jun 4;17:100501. doi: 10.1016/j.abrep.2023.100501 (PMC10279774; doi:10.1016/j.abrep.2023.100501)
Supplement: Supplementary Data 1 [file mmc1.docx]

**Supplementary Material - Figures:**

| eFigure 1. Temporal order of pairwise disorders | eFigure 2. Temporal order of pairwise disorders: males only | eFigure 3. Temporal order of pairwise disorders: females only |
| --- | --- | --- |
| 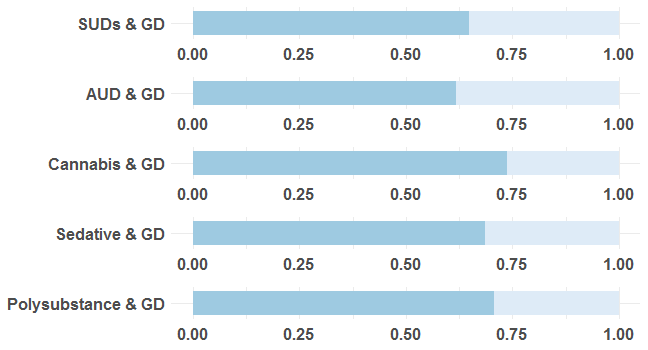 | 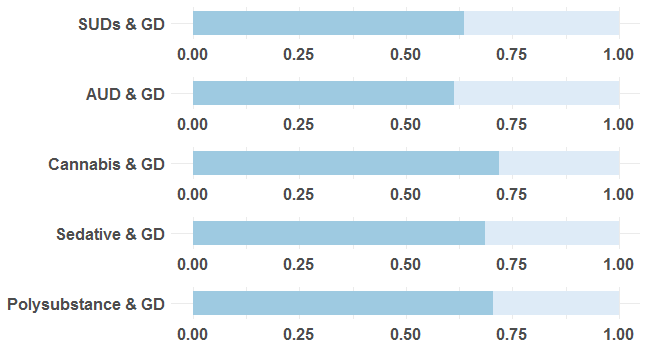 | 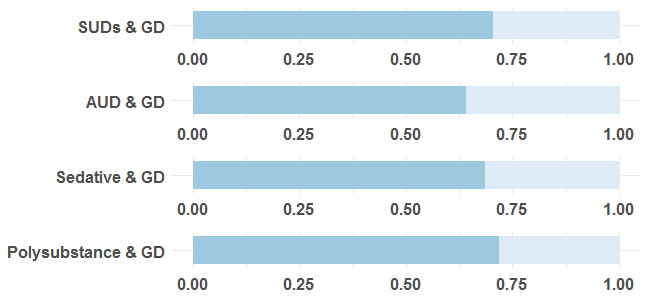 |

*Note*: The proportion of cases where SUD is the first diagnosed disorder is represented by the dark blue bars for each pairwise disorder. The light blue bars represent the proportion of cases where SUD is the second diagnosed disorder. Co-occurring cases were removed from analysis of temporal order and are not included here; however, they are presented in the Supplementary Material. Given the low n for cannabis use in females (i.e., 10 patients only), it is not represented in Figure 3.

| eFigure 4. Temporal order of pairwise disorders: with 12 month lag | eFigure 5. Temporal order of pairwise disorders: males only with 12 month lag | eFigure 6. Temporal order of pairwise disorders: females only with 12 month lag |
| --- | --- | --- |
| 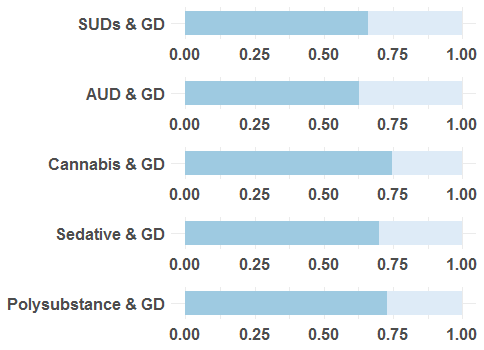 | 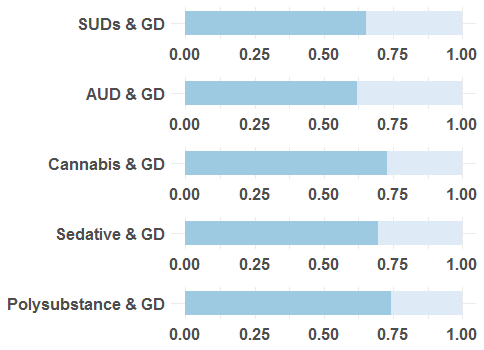 | 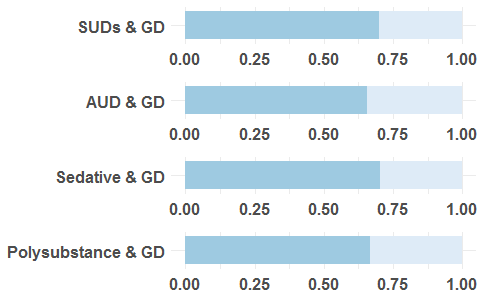 |

*Note*: The proportion of cases where SUD is the first diagnosed disorder is represented by the dark blue bars for each pairwise disorder. The light blue bars represent the proportion of cases where SUD is the second diagnosed disorder. Co-occurring cases were removed from analysis of temporal order and are not included here; however, they are presented in the supplementary material. Given the low n for cannabis use in females (i.e., 10 patients only), it is not represented in Figure 6.

| eFigure 7. Temporal order of pairwise disorders | eFigure 8. Temporal order of pairwise disorders: males only | eFigure 9. Temporal order of pairwise disorders: females only |
| --- | --- | --- |
| 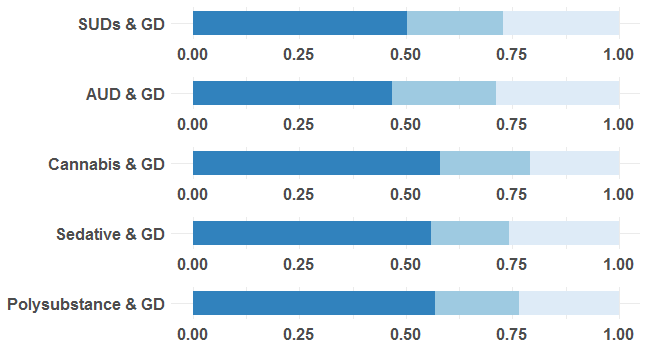 | 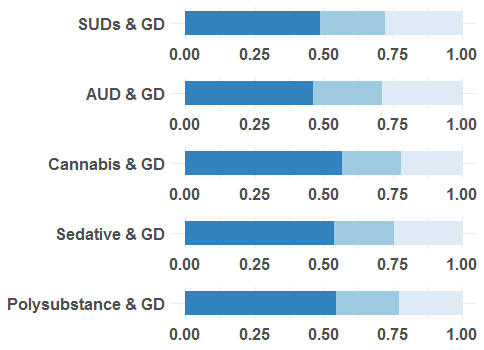 | 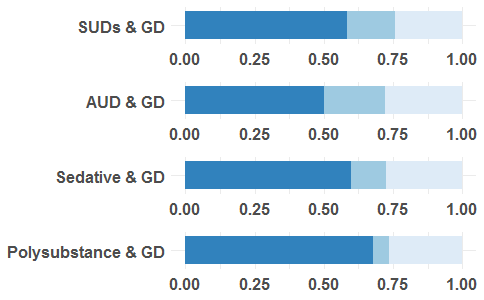 |

Note: All cases included. The proportion of cases where SUD is the first diagnosed disorder is represented by the dark blue bars for each pairwise disorder. The lighter blue bars represent the proportion of cases where SUD and GD were diagnosed within the same month. The lightest blue bars represent GD as the first diagnosed disorder. Given the low n for cannabis use in females (i.e., 12 patients only), it is not represented in Figure 3.

| eFigure 10. Temporal order of pairwise disorders: with 12-month lag | eFigure 11. Temporal order of pairwise disorders: males only with 12-month lag | eFigure 12. Temporal order of pairwise disorders: females only with 12-month lag |
| --- | --- | --- |
| 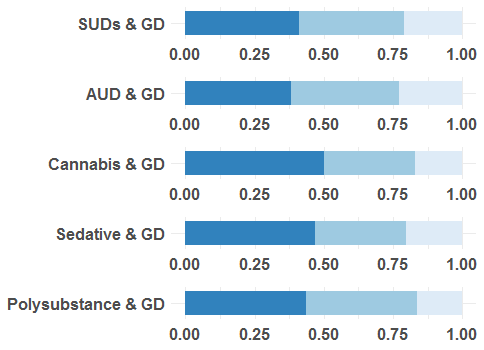 | 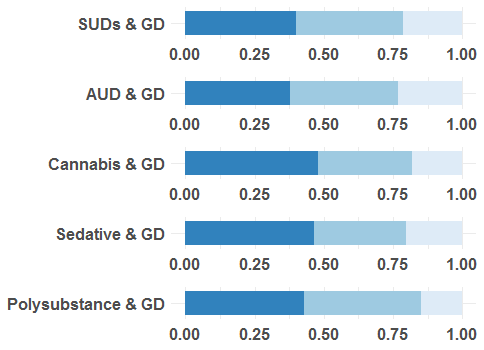 | 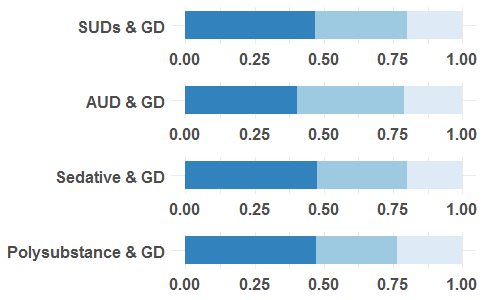 |

Note: All cases included. The proportion of cases where SUD is the first diagnosed disorder is represented by the dark blue bars for each pairwise disorder. The lighter blue bars represent the proportion of cases where SUD and GD were diagnosed within the same month. The lightest blue bars represent GD as the first diagnosed disorder. Given the low n for cannabis use in females (i.e., 12 patients only), it is not represented in Figure 3.
